# Oral health conditions in patients under antiresorptive therapy are comparable to unexposed during supportive periodontal care

**DOI:** 10.1007/s00784-023-05257-y

**Published:** 2023-09-15

**Authors:** Antonio Ciardo, Marlinde M. Simon, Sinclair Awounvo, Ti-Sun Kim

**Affiliations:** 1 Section of Periodontology, Department of Conservative Dentistry, Clinic for Oral, Dental and Maxillofacial Diseases, Heidelberg University Hospital, Im Neuenheimer Feld 400, 69120 Heidelberg, Germany; 2 Institute of Medical Biometry, University of Heidelberg, Heidelberg, Germany

**Keywords:** Periodontitis, Medication-related osteonecrosis of the jaw, Bisphosphonates, Denosumab, Preventive dentistry, Oral health-related quality of life

## Abstract

**Objectives:**

To investigate oral health and oral health-related quality of life (OHRQoL) of patients under antiresorptive therapy (ART) during supportive periodontal care (SPC) considering history of medication-related osteonecrosis of the jaw (MRONJ).

**Materials and methods:**

In this cross-sectional study, 100 patients (50 receiving ART (exposed) and 50 without ART (unexposed)) in regular SPC were enrolled for a clinical oral examination and the evaluation of OHRQoL using the OHIP-G14-questionnaire. History of MRONJ was assessed by anamnesis and reviewing patient records.

**Results:**

There were no statistically significant group differences in age (exposed: 70.00 ± 9.07 versus unexposed: 71.02 ± 8.22 years), sex, distribution of systemic diseases and duration of SPC (on average 8.61 ± 5.73 years). Number of teeth (21.02 ± 5.84 versus 21.40 ± 5.42), DMFT (18.38 ± 3.85 versus 17.96 ± 4.08), probing pocket depth (2.31 ± 0.20 versus 2.38 ± 0.26), clinical attachment level (3.25 ± 0.76 versus 3.46 ± 0.58) and bleeding on probing (15.07 ± 11.53 versus 15.77 ± 13.08) were also not significantly different. The OHIP-G14 sum-score was significantly higher in exposed participants (6.10 ± 6.76 versus 3.62 ± 5.22, *p* = 0.043). History of MRONJ was prevalent in 8% of patients under ART. Periodontal/peri-implant-related MRONJ were reported in three participants with cancer (n = 1 before and n = 2 after active periodontal therapy). History of MRONJ due to endodontic/restorative reasons was reported in one patient with osteoporosis.

**Conclusions:**

Patients under ART in SPC demonstrated similar clinical periodontal and dental status but lower OHRQoL compared to unexposed (not statistically significant). Patient awareness of the MRONJ-risk and appropriate preventive measures should be ensured.

**Clinical relevance:**

SPC in osteoporotic patients under ART appeared safe regarding MRONJ, but further investigations on the MRONJ-risk in patients with different risk-profiles are necessary.

**Study registration**: clinicaltrials.gov (#NCT04192188).

**Supplementary Information:**

The online version contains supplementary material available at 10.1007/s00784-023-05257-y.

## Introduction

Antiresorptives, such as bisphosphonates (BPs) or monoclonal antibodies as denosumab (DNO), are prescribed to patients with osteoporosis or tumor diseases, for example breast or prostate carcinoma, and also in prevention or in cases of bone metastases [1–4]. Their pharmacological effect is based on intervening in the bone metabolism and suppressing osteoclast activity, albeit with different mechanisms of action [5]. BPs accumulate in bone by selectively binding to hydroxyapatite [5]. They inactivate intracellulary various cell types, in particular, osteoclasts [5]. DNO though, binds selectively to the RANK ligand and thereby inhibits the development, activation, and survival of osteoclasts [5, 6]. A complication resulting from ART is medication-related osteonecrosis of the jaw (MRONJ) [7]. The pathogenesis is controversial, but one pathway is widely accepted: local inflammatory processes of the alveolar bone or physiological bone remodeling after tooth extraction are dependent on osteoclast activity, or otherwise can lead to osteonecrosis [6, 8]. Due to their pharmacokinetics, BPs exhibit a cumulative effect [5].

For dentists and maxillofacial surgeons, MRONJ represent a major challenge in prevention, diagnosis and therapy [9]. MRONJ have a lasting impact on the quality of life of affected patients [10].

Patients receiving antiresorptives are often unaware of the MRONJ-risk and appropriate preventive strategies [11]. Risk factors for the occurrence of MRONJ, besides infectious diseases of the alveolar bone, such as periodontitis, include poor oral hygiene and poorly fitting dental prostheses, but also tooth extractions or oral surgery during ART [12]. Hallmer et al. detected anaerobic bacteria representative of periodontal microflora, mainly *Porphyromonas, Lactobacillus, Tannerella, Prevotella, Actinomyces, Treponema, Streptococcus and Fusobacterium* in MRONJ sites using 16S rRNA sequencing [13]. For the therapy of periodontal disease and at the same time for the prevention of MRONJ, tooth extractions or conventional subgingival instrumentation can be performed respecting necessary precautions, such as the administration of antibiotics [12, 14, 15].

The extent to which periodontal treatment has an influence on the development or prevention of MRONJ in patients receiving ART is not established. A case report shows MRONJ as a consequence of periodontal therapy [16]. There are no scientific data on patients under ART in supportive periodontal care (SPC) after active periodontal therapy. Such data are needed to enable an evidence-based decision-making and to reduce extractions of periodontally compromised teeth, which themselves represent a MRONJ-risk factor, to a necessary level, while safely providing periodontal therapy in relation to the MRONJ-risk. There is also no evidence to what extent oral health or oral health-related quality of life (OHRQoL) in periodontal maintenance therapy differ in patients with and without history of antiresorptive medication.

Hence, the aim of this study was primarily to investigate to which extent antiresorptive medication associates to oral health, objectively by number of teeth, periodontal status, DMFT and DMFS as well as subjectively, by means of oral health-related quality of life (OHRQoL) in a cohort of elderly adults during SPC. The secondary research questions were whether patients receiving ART 1) were informed of their risk of MRONJ, 2) had a history of MRONJ, and if so, 3) which were the reasons for MRONJ.

## Materials and methods

This cross-sectional study was approved by the ethics committee of the Medical Faculty of Heidelberg (# S-630/2019) and is in accordance with the ethical standards of the 1964 Helsinki Declaration and its later amendments or comparable ethical standards. The study was registered at the US National Institute of Health (ClinicalTrials.gov, #NCT04192188). Written informed consent was obtained from all individuals included in the study. This study is being reported using the “Strengthening the Reporting of Observational studies in Epidemiology” (STROBE) guidelines [17].

### Study sample and setting

**Fig. 1:**
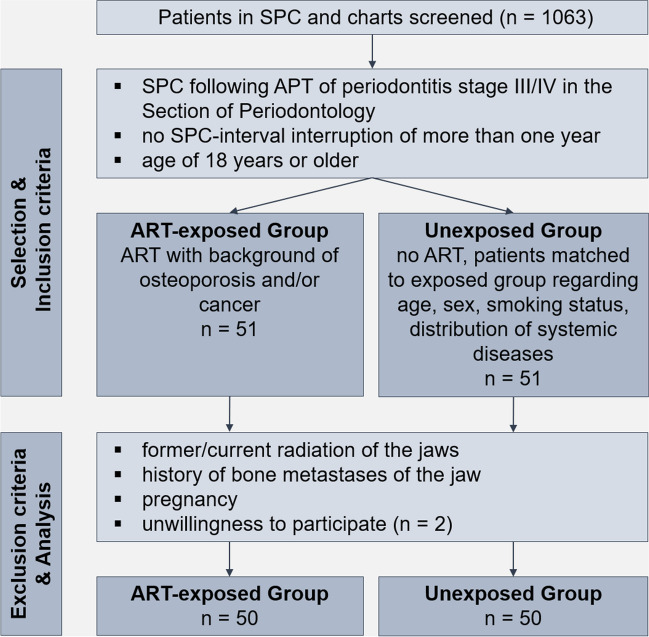
Flow chart study participant selection. Abbreviations: SPC = supportive periodontal care, APT = active periodontal therapy, n = patients, ART = antiresorptive therapy

One hundred study participants were recruited during routine dental care in the Section of Periodontology of the Department of Conservative Dentistry at Heidelberg University Hospital between December 2019 and February 2021 for a single study visit. 1063 charts and patients in SPC were screened. Inclusion criteria were patients of 18 years or older undergoing regular SPC (no interval interruption of more than one year) following active periodontal therapy of periodontitis stage III/IV [18] in the past in our department. The exposed group included patients under antiresorptive drugs within the last ten years with a background of osteoporosis or cancer. The unexposed participants were recruited as a comparative patient collective without antiresorptive medication but frequency matched to the exposed group in terms of age, sex, smoking status, and distribution of systemic diseases. For frequency matching, exposed participants were generally recruited first. Exclusion criteria were former/current radiation or metastases of the jaws and pregnancy (Fig. 1).

In general, routine SPC sessions in our department were carried out in a standardized manner. The frequency of SPC was dependent on the periodontitis risk assessment according to Ramseier and Lang [19]. In most cases, SPC took place every six months. Dental hygienists or dental students performed a professional supragingival tooth cleaning and motivated patients to practice adequate oral hygiene. They recommended the use of individually correctly fitting interdental brushes. Diagnostics were performed as described in “Clinical examination” by calibrated dentists of the Section of Periodontology. Teeth with probing pocket depths (PPD) of 5 mm or greater or 4 mm with bleeding on probing (BOP) were reinstrumented with Gracey curettes and sonic tips. In patients with deeper pockets of 6 mm or more, either reinstrumentation or periodontal surgical treatment was performed. However, in patients on ART, the indication for periodontal surgery is strict.

### Clinical examination

Before oral examination, all participants filled out a survey covering personal information, medical history, antiresorptive medication and oral health-related measures, such as smoking history.

The clinical examination was part of a routine SPC session and included the following standardized procedures.

A dental hygienist collected the Gingival Bleeding Index, GBI [20] and the Plaque Control Record, PCR [21], and performed a professional supragingival tooth cleaning.

The oral examination was performed by a single dentist specialized in periodontology (AC) in a standardized manner and included the inspection of the oral mucosa for pathologies, especially dehiscences indicating MRONJ. The assessment of the number of teeth and implants, and the number and extent of carious lesions and restorations by tactile inspection of the teeth with a probe (EXS3A6, Hu-Friedy Mfg. Co., LLC., Frankfurt/Main, Germany). Existing removable prostheses were checked for function and sharp edges. Pulp testing and sensitivity to percussion testing have been performed for endodontic evaluation. The findings were documented manually on a sheet and the DMFT and DMFS indeces were recorded digitally afterwards. A full periodontal status included the measurements of PPD at six sites per tooth or implant using a periodontal probe (PCPUNC15, Hu-Friedy, Frankfurt/Main, Germany). The probe was carefully inserted into the gingival or peri-implant sulcus to the bottom of the pocket with a force of approximately 0.2 N. The clinical attachment level (CAL) was recorded simultaneously and was defined as the measured distance from the bottom of the pocket to the cementoenamel junction. If the cementoenamel junction was not detectable due to restorations, the restoration margin served as the coronal reference point. In the case of implants, no clinical attachment level was measured. BOP was assessed and defined as bleeding after PPD/CAL measurements. Tooth mobility was recorded in three degrees: 0 = physiological tooth mobility, I = tooth mobility ≤ 1 mm, II = tooth mobility ≤ 2 mm, III = vertical or lateral tooth mobility > 2 mm [22]. Furcation involvements were measured with a Nabers probe (#2N Gr. 31, Hu-Friedy, Frankfurt/Main, Germany) distinguishing three grades: I = furcation ≤ 3 mm, II = furcation > 3 mm and III = continuous furcation measurable [23]. Periodontal data were recorded digitally (ParoStatus.de GmbH, Berlin, Germany).

### History of MRONJ and OHRQoL

Panoramic radiographs were obtained independently of the study depending on clinical indication or generally every five years during SPC. If MRONJ were suspected [24], standard procedure in our department was to refer patients to a maxillofacial surgeon and expert in MRONJ for further radiological diagnostics, diagnosis confirmation and further treatment of possible MRONJ. For this investigation, history of MRONJ was obtained through medical history and confirmed by review of patient records.

In order to assess knowledge/experience of patients concerning ART, five yes–no questions were asked: 1) “Are you aware that osteonecrosis of the jaw is a possible side effect of antiresorptive medication?”, 2) “Were you referred to a dentist or maxillofacial surgeon for an oral check-up prior to taking or receiving antiresorptive medication?”, 3) “Did you have any teeth removed as a precaution before taking or receiving antiresorptive medication?”, 4) “Were you informed about oral preventive measures while taking or receiving antiresorptive medication?”, 5) “Would you be willing to implement additional oral preventive measures while taking or receiving antiresorptive medication?”.

OHRQoL was assessed by means of the German version of the 14-item Oral Health Impact Profile (OHIP-G14). The answers were recorded on a Likert scale with values ranging from 0 to 4 coded as 0 “never”, 1 “hardly ever”, 2 “occasionally”, 3 “fairly often”, or 4 “very often”. The OHIP-G14 sum score can range from 0 to 56 with a higher score indicating poorer OHRQoL [25]. The 14 items can be grouped into seven sub-domains: 1) functional limitation (items 1 and 2), 2) handicap (3 and 10), 3) psychological disability (4 and 11), 4) psychological discomfort (5 and 14), 5) physical disability (6 and 12), 6) physical pain (7 and 13), and 7) social disability (8 and 9).

### Sample size and statistical analysis

In order to perform a sample size calculation, a potential clinically relevant mean CAL difference (primary objective) of 1.00 ± 1.75 mm was assumed for both exposed and unexposed patients [26]. Under these assumptions, 50 participants per group were needed to detect an effect with a power of 1-β = 80% as assumed above, using a two-sample t-test with assumption of equal variance to the two-sided significance level of α = 5%. The power calculation was performed using PASS 16.0.3 software.

Patients’ demographic and clinical examination data were assessed descriptively. Continuous variables were described using the number of non-missing values, mean, standard deviation, median, Q1, Q3, minimum and maximum. For categorical variables absolute and relative frequencies were provided.

In addition, a two-sided Welch’s two-sample t-test and homogeneity tests (Pearson’s Chi-squared test of homogeneity or Boschloo’s test) were performed for continuous and categorical variables, respectively, to determine whether the exposed and unexposed group significantly differed regarding the variable of interest at the 5% level. P-values were reported alongside 95% confidence intervals for the mean difference and the proportion difference.

With the exception of the test for the mean difference in CAL between the two groups, all other p-values were interpreted descriptively.

A linear regression analysis with stepwise variable selection (forward and backward) using AIC was conducted for the outcomes CAL, PPD, BOP and OHIP-G14 sum score to investigate whether and how they were influenced by some factors. Regression analyses were performed at patient level using the mean outcome as dependent variable. All models included at least “group (exposed, unexposed)”, “age”, “diabetes mellitus (yes, no)” and “smoking (pack years)” as independent variables. Additional variables such as “sex (male, female)”, “duration of SPC” and “PCR” were also investigated for the outcomes CAL, PPD and BOP. For the outcome OHIP-G14 sum score, the variables “MRONJ history (yes, no)”, “DMFT”, “DMFS”, “need for subgingival instrumentation (PPD ≥ 5 mm or 4 mm + BOP; yes, no)”, “implants (yes, no)” and “removable dentures (no, tooth-/implant-supported, mucosally supported)” were also part of the variable selection procedure. The outcomes CAL and PPD were log-transformed and BOP and OHIP-G14 sum score square-root-transformed to satisfy the normality assumption of the residuals.

Statistical analyses were performed using R version 4.1.1 and carried out at the Institute for Medical Biometry (IMBI) at the University of Heidelberg.

## Results

### Patients’ demographic data

The study population consisted of a total of 100 patients, 50 (50%) ART-exposed and unexposed, respectively, of whom 75 were women and 25 were men, with no statistically significant differences between the two groups (*p* = 0.271^Bolo^). On average, participants were 70.51 ± 8.63 years old (*p* = 0.557^tt2^) and took part in SPC for a total of 8.61 ± 5.73 years with no statistically significant group differences (*p* = 0.557^tt2^; *p* = 0.665^tt2^). While the distribution of smoking status (never, former, current) showed statistically no significant difference (*p* = 0.084^chi2^), the calculated pack years appeared significantly higher in the unexposed group with 11.27 ± 15.36 compared to 5.53 ± 11.84 pack years in the exposed group (*p* = 0.039^tt2^). On average, study participants had 3.14 ± 1.49 known systemic diseases with statistically significant difference between the exposed (3.60 ± 1.60) and unexposed group (2.68 ± 1.22, *p* = 0.002^tt2^). However, the categorical distribution of the number of systemic diseases was not statistically significantly different (*p* = 0.153). Osteoporosis, cancer and rheumatoid disease were statistically significantly more prevalent in the exposed than in the unexposed group. The reason for ART was osteoporosis in 36 study participants (72%), cancer or bone metastases in 13 (26%), and a combination of osteoporosis and cancer in one case (2%). In 16 cases (32%), antiresorptives were administered orally, in 32 cases (64%) intravenously/subcutaneously, and two participants (4%) received both administration forms. 49 patients (98%) received BPs and nine (18%) DNO, leaving eight participants receiving both antiresorptives (Table 1).

**Table 1:** Participants’ characteristics

Variables	Total (*n* = 100)	Unexposed Group (*n* = 50)	ART-exposed Group (*n* = 50)	*p*-value	Confidence Interval
Sex					
Male	25 (25%)	15 (30%)	10 (20%)	0.271^Bolo^	Prop. dif. CI [-0.089, 0.36]
Female	75 (75%)	35 (70%)	40 (80%)		
Age (years)					
Mean ± SD	70.51 ± 8.63	71.02 ± 8.22	70.00 ± 9.07	0.557^tt2^	Mean dif. CI [-2.4, 4.5]
Median (Q1 – Q3)	72.00 (65.00 – 77.00)	72.00 (64.00 – 77.00)	71.50 (65.00 – 76.00)		
Min – Max	41 – 89	53 – 88	41 – 89		
Number of systemic diseases					
0	1 (1%)	1 (2%)	0 (0%)	0.153^chi2^	
1	10 (10%)	6 (12%)	4 (8%)		
2	26 (26%)	17 (34%)	9 (18%)		
3	28 (28%)	15 (30%)	13 (26%)		
4	18 (18%)	7 (14%)	11 (22%)		
5	9 (9%)	3 (6%)	6 (12%)		
6	5 (5%)	1 (2%)	4 (8%)		
7	3 (3%)	0 (0%)	3 (6%)		
Number of systemic diseases					
Mean ± SD	3.14 ± 1.49	2.68 ± 1.22	3.60 ± 1.60	**0.002**^**tt2**^	Mean dif. CI [-1.5, -0.35]
Median (Q1 – Q3)	3.00 (2.00 – 4.00)	3.00 (2.00 – 3.00)	3.00 (2.00 – 5.00)		
Min – Max	0 – 7	0 – 6	1 – 7		
Heart disease					
Yes	33 (33%)	17 (34%)	16 (32%)	0.910^Bolo^	Prop. dif. CI [-0.23, 0.19]
Hypertension					
Yes	58 (58%)	33 (66%)	25 (50%)	0.115^Bolo^	Prop. dif. CI [-0.36, 0.03]
Apoplexy					
Yes	7 (7%)	4 (8%)	3 (6%)	0.910^Bolo^	Prop. dif. CI [-0.46, 0.3]
Epilepsy					
Yes	3 (3%)	1 (2%)	2 (4%)	0.786^Bolo^	Prop. dif. CI [-0.37, 0.71]
Renal disease					
Yes	9 (9%)	3 (6%)	6 (12%)	0.370^Bolo^	Prop. dif. CI [-0.14, 0.51]
Pulmonary disease					
Yes	15 (15%)	8 (16%)	7 (14%)	0.860^Bolo^	Prop. dif. CI [-0.31, 0.23]
Gastro-intestinal disease					
Yes	8 (8%)	3 (6%)	5 (10%)	0.617^Bolo^	Prop. dif. CI [-0.21, 0.49]
Glaucoma					
Yes	11 (11%)	4 (8%)	7 (14%)	0.420^Bolo^	Prop. dif. CI [-0.15, 0.46]
Hepatic disease					
Yes	5 (5%)	4 (8%)	1 (2%)	0.271^Bolo^	Prop. dif. CI [-0.68, 0.05]
Osteoporosis					
Yes	57 (57%)	13 (26%)	44 (88%)	** < 0.001**^**Bolo**^	Prop. dif. CI [0.48, 0.78]
Rheumatoid disease					
Yes	19 (19%)	5 (10%)	14 (28%)	**0.024**^**Bolo**^	Prop. dif. CI [0.07, 0.52]
Thyroid disease					
Yes	42 (42%)	19 (38%)	23 (46%)	0.458^Bolo^	Prop. dif. CI [-0.12, 0.28]
Diabetes mellitus					
Yes	11 (11%)	7 (14%)	4 (8%)	0.420^Bolo^	Prop. dif. CI [-0.46, 0.15]
Cancer					
Yes	36 (36%)	13 (26%)	23 (46%)	**0.039**^**Bolo**^	Prop. dif. CI [0.02, 0.42]
Smoking status					
Never	57 (57%)	23 (46%)	34 (68%)	0.084^chi2^	
Former	38 (38%)	24 (48%)	14 (28%)		
Current	5 (5%)	3 (6%)	2 (4%)		
Smoking (pack years)					
Mean ± SD	8.40 ± 13.95	11.27 ± 15.36	5.532 ± 11.84	**0.039**^**tt2**^	Mean dif. CI [0.29, 11]
Median (Q1 – Q3)	0 (0 – 12.25)	2.81 (0 – 23.75)	0 (0 – 6.000)		
Min – Max	0 – 60	0 – 56	0 – 60		
Duration of SPC (years)					
Mean ± SD	8.61 ± 5.73	8.86 ± 5.88	8.36 ± 5.63	0.665^tt2^	Mean dif. CI [-1.8, 2.8]
Median (Q1 – Q3)	9.00 (3.00 – 14.00)	9.50 (3.00 – 14.00)	8.50 (3.00 – 13.00)		
Min – Max	0 – 19	0 – 19	0 – 18		
Underlying disease for ART					
Osteoporosis			36 (72%)		
Cancer			8 (16%)		
Bone metastasis			5 (10%)		
Osteoporosis and cancer			1 (2%)		
ART administration form					
Oral			16 (32%)		
Intravenous/subcutaneous			32 (64%)		
Oral and intravenous/subcutaneous			2 (4%)		
Antiresorptives					
Bisphosphonates			49 (98%)		
Denosumab			9 (18%)		
One antiresorptive drug			42 (84%)		
Two antiresorptive drugs			8 (16%)		
MRONJ history					
No			46 (92%)		
Yes			4 (8%)		

Abbreviations: *n* number of participants, *SD* standard deviation, *Q* quartile, *Min* Minimum, *Max* Maximum, *dif.* difference, *CI* Confidence Interval, ^*Bolo*^ Boschloo’s test, ^*tt2*^ Welch’s two-sample t-test, ^*chi2*^ Pearson’s chi-squared test, *ART* antiresorptive therapy, *SPC* supportive periodontal care, *MRONJ* medication-related osteonecrosis of the jaw. Significant values are in bold

**Fig. 2:**
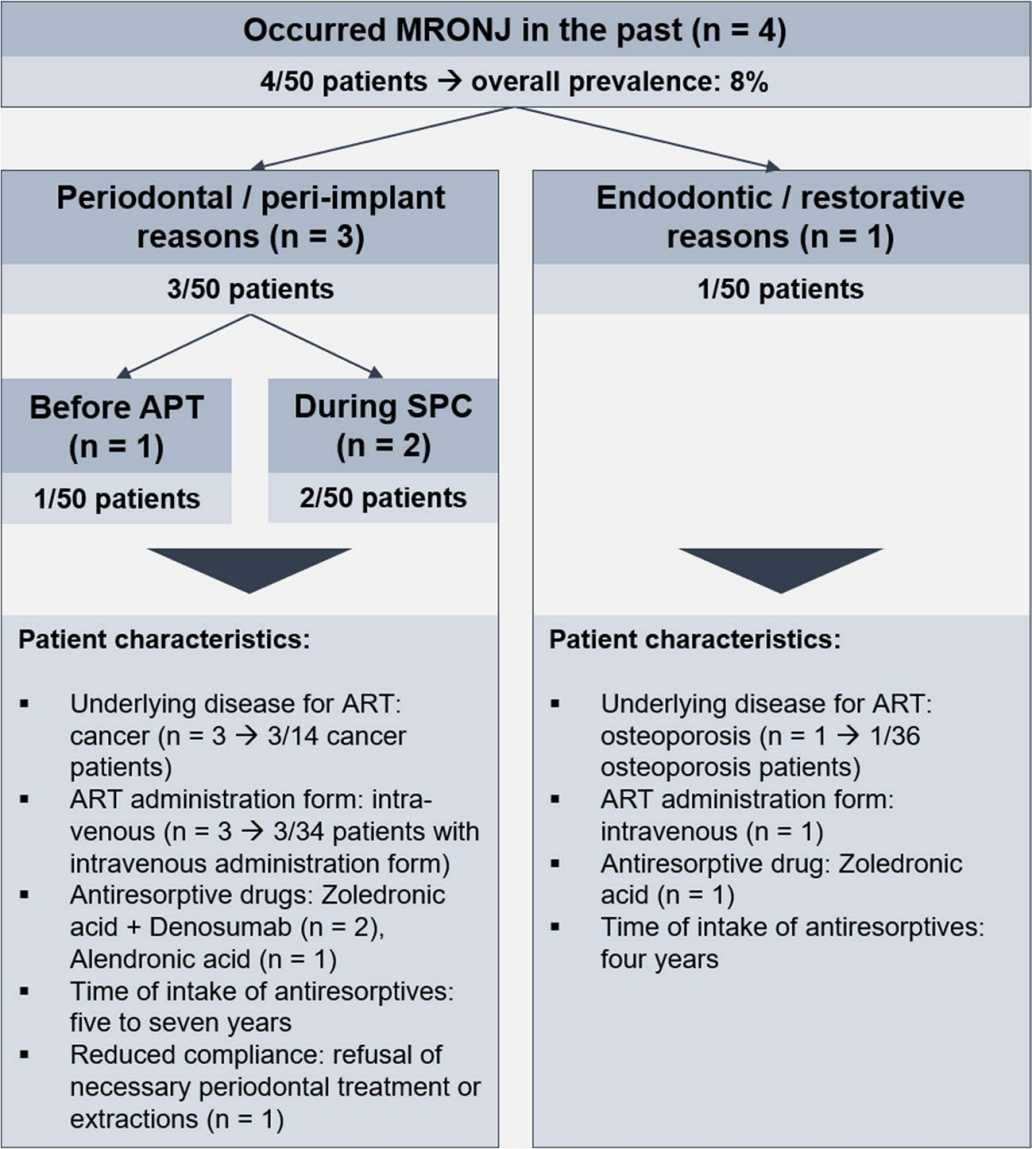
Occurred MRONJ and patients’ characteristics. Abbreviations: MRONJ = medication-related osteonecrosis of the jaw, n = patients, APT = active periodontal therapy, SPC = supportive periodontal care, ART = antiresorptive therapy

In the exposed group, four participants (8%) had a history of MRONJ either due to periodontal/peri-implant (*n* = 3) or endodontic/restorative reasons (*n* = 1). Among those three patients with MRONJ due to periodontal reasons, one suffered from MRONJ before and two after active periodontal therapy. All three participants had cancer as underlying condition for ART and received antiresorptives intravenously/subcutaneously for five to seven years. Two of them received BPs and additionally DNO (Fig. 2). Overall, prevalences of MRONJ in the exposed group amounted to 8%. The two patients affected by MRONJ during SPC, were not exposed to additional risk factors, such as periodontal surgery, and did not adhere to recommended tooth extractions, as was evident from the patients' medical records.

### Clinical findings

In this patient cohort 21.21 ± 5.61 teeth were present without statistically significant difference between the exposed (21.40 ± 5.42) and unexposed group (21.02 ± 5.84, *p* = 0.737^tt2^). The DMFT index was 18.17 ± 3.95 overall without statistically significant difference between the exposed (18.38 ± 3.85) and unexposed group (17.96 ± 4.08, *p* = 0.598^tt2^). Similarly, the DMFS index was 67.43 ± 20.47 in the total sample, 67.46 ± 20.72 in the exposed and 67.40 ± 20.42 in the unexposed group (*p* = 0.988^tt2^). This was also true for the respective subcategories “Decayed”, “Missing” and “Filled”. Removable dentures were present in 22 patients, while tooth- or implant-supported dentures were present in 16 and mucosally supported prostheses in six patients without statistically significant differences between the two groups (*p* = 0.571^chi2^).

GBI was 4.28% ± 5.41% and PCR 33.66% ± 15.51% overall with no statistically significant group differences (*p* = 0.532^tt2^ and *p* = 0.324^tt2^, respectively).

Mean CAL was 3.36 ± 0.68 mm overall, 3.25 ± 0.76 mm in the exposed group and 3.46 ± 0.58 mm in the unexposed group (*p* = 0.117^tt2^). Mean PPD were in total 2.35 ± 0.24 mm, 2.31 ± 0.20 mm (exposed) and 2.38 ± 0.26 mm (unexposed, *p* = 0.093^tt2^). BOP was also not statistically significantly different at 15.42% ± 12.27% (total), 15.07% ± 11.53% (exposed) and 15.77% ± 13.08% (unexposed, *p* = 0.788^tt2^). Manifestation of furcation involvement was not statistically significantly different between the four grades 0–3. For tooth mobility however, statistically significant differences related to the classification of teeth with grade 0 (*p* = 0.023^tt2^) with 85.00% ± 13.72% (exposed) versus 90.44% ± 9.29% (unexposed) as well as with grade 1 (*p* = 0.011^tt2^) with 14.19% ± 13.25% (exposed) versus 8.56% ± 7.70% (unexposed) have been evaluated.

Further, there were no significant differences in the number of teeth in need for subgingival reinstrumentation, namely with PPD of 5 mm or greater or 4 mm with BOP (2.20 ± 2.19 (exposed) versus 1.92 ± 2.10 (unexposed), *p* = 0.515^tt2^, Table 2).

**Table 2:** Clinical findings

Variables	Total (*n* = 100)	Unexposed Group (*n* = 50)	ART-exposed Group (*n* = 50)	*p*-value	Confidence Interval
Number of teeth					
Mean ± SD	21.21 ± 5.61	21.02 ± 5.84	21.40 ± 5.42	0.737^tt2^	Mean dif. CI [-2.6, 1.9]
Median (Q1 – Q3)	23.00 (18.00 – 26.00)	23.00 (19.00 – 26.00)	22.50 (18.00 – 26.00)		
Min – Max	6 – 31	6 – 28	9 – 31		
Number of implants					
Mean ± SD	0.91 ± 1.94	1.26 ± 1.94	0.56 ± 1.90	0.071^tt2^	Mean dif. CI [-0.061, 1.5]
Median (Q1 – Q3)	0 (0 – 1.0)	0 (0 – 2.0)	0 (0 – 0)		
Min – Max	0 – 10	0 – 8	0 – 10		
DMFT					
Mean ± SD	18.17 ± 3.95	17.96 ± 4.08	18.38 ± 3.85	0.598^tt2^	Mean dif. CI [-2, 1.2]
Median (Q1 – Q3)	18.00 (16.00 – 21.00)	18.00 (15.00 – 20.00)	18.00 (16.00 – 21.00)		
Min – Max	8 – 26	8 – 25	10 – 26		
Decayed teeth					
Mean ± SD	0.22 ± 0.58	0.12 ± 0.48	0.32 ± 0.65	0.084^tt2^	Mean dif. CI [-0.43, 0.028]
Median (Q1 – Q3)	0 (0 – 0)	0 (0 – 0)	0 (0 – 0)		
Min – Max	0 – 3	0 – 3	0 – 3		
Missing teeth					
Mean ± SD	7.30 ± 5.35	7.60 ± 5.69	7.00 ± 5.03	0.577^tt2^	Mean dif. CI [-1.5, 2.7]
Median (Q1 – Q3)	6.00 (3.00 – 10.00)	6.00 (3.00 – 9.00)	6.00 (2.00 – 10.00)		
Min – Max	0 – 22	1 – 22	0 – 19		
Filled teeth					
Mean ± SD	10.65 ± 5.12	10.24 ± 4.96	11.06 ± 5.30	0.426^tt2^	Mean dif. CI [-2.9, 1.2]
Median (Q1 – Q3)	11.50 (7.00 – 14.00)	12.00 (6.00 – 14.00)	11.00 (7.00 – 15.00)		
Min – Max	0 – 23	0 – 23	1 – 23		
DMFS					
Mean ± SD	67.43 ± 20.47	67.40 ± 20.42	67.46 ± 20.72	0.988^tt2^	Mean dif. CI [-8.2, 8.1]
Median (Q1 – Q3)	67.00 (53.50 – 81.50)	67.50 (55.00 – 79.00)	67.00 (52.00 – 82.00)		
Min – Max	18 – 115	18 – 112	27 – 115		
Decayed surfaces					
Mean ± SD	0.25 ± 0.72	0.16 ± 0.74	0.34 ± 0.69	0.210^tt2^	Mean dif. CI [-0.46, 0.1]
Median (Q1 – Q3)	0 (0 – 0)	0 (0 – 0)	0 (0 – 0)		
Min – Max	0 – 5	0 – 3	0 – 5		
Missing surfaces					
Mean ± SD	34.91 ± 24.86	36.28 ± 26.24	33.54 ± 23.60	0.584^tt2^	Mean dif. CI [-7.2, 13]
Median (Q1 – Q3)	30.00 (15.00 – 48.00)	30.00 (15.00 – 44.00)	29.00 (10.00 – 48.00)		
Min – Max	0 – 103	5 – 103	0 – 88		
Filled surfaces					
Mean ± SD	32.27 ± 18.26	30.96 ± 17.64	33.58 ± 18.96	0.476^tt2^	Mean dif. CI [-9.9, 4.6]
Median (Q1 – Q3)	32.50 (17.50 – 45.00)	32.50 (16.00 – 44.00)	32.00 (18.00 – 45.00)		
Min – Max	0 – 79	0 – 63	2 – 79		
Removable dentures					
No	78 (78%)	37 (74%)	41 (82%)	0.571^chi2^	
Yes, tooth/implant-supported	16 (16%)	9 (18%)	7 (14%)		
Yes, mucosally supported	6 (6%)	4 (8%)	2 (4%)		
Gingival Bleeding Index [%] †					
Mean ± SD	4.28 ± 5.41	3.94 ± 5.38	4.62 ± 5.47	0.532^tt2^	Mean dif. CI [-2.8, 1.5]
Median (Q1 – Q3)	2.00 (0.50 – 6.00)	2.00 (0 – 5.00)	3.00 (1.00 – 7.00)		
Min – Max	0 – 27	0 – 24	0 – 27		
Plaque Control Record [%] ‡					
Mean ± SD	33.66 ± 15.51	32.12 ± 18.15	35.20 ± 12.33	0.324^tt2^	Mean dif. CI [-9.2, 3.1]
Median (Q1 – Q3)	32.50 (22.00 – 42.00)	29.00 (19.00 – 42.00)	36.00 (27.00 – 43.00)		
Min – Max	3 – 79	8 – 79	3 – 58		
Clinical attachment level [mm]					
Mean ± SD	3.36 ± 0.68	3.46 ± 0.58	3.25 ± 0.76	0.117^tt2^	Mean dif. CI [-0.05, 0.48]
Median (Q1 – Q3)	3.31 (2.85 – 3.73)	3.43 (3.02 – 3.77)	3.185 (2.69 – 3.63)		
Min – Max	0–15	1–11	0–15		
Probing pocket depth [mm]					
Mean ± SD	2.35 ± 0.24	2.38 ± 0.26	2.31 ± 0.20	0.093^tt2^	Mean dif. CI [-0.01, 0.17]
Median (Q1 – Q3)	2.30 (2.19 – 2.47)	2.30 (2.21 – 2.57)	2.30 (2.18 – 2.43)		
Min – Max	1–15	1–9	1–15		
Bleeding on probing [%]					
Mean ± SD	15.42 ± 12.27	15.77 ± 13.08	15.07 ± 11.53	0.778^tt2^	Mean dif. CI [-4.2, 5.6]
Median (Q1 – Q3)	12.92 (7.50 – 20.33)	13.14 (7.05 – 22.44)	12.78 (7.64 – 20.00)		
Min – Max	0 – 73	0 – 73	0 – 62		
Furcation Grade 0 [%] §					
Mean ± SD	81.03 ± 12.57	80.47 ± 12.81	81.60 ± 12.42	0.654^tt2^	Mean dif. CI [-6.1, 3.9]
Median (Q1 – Q3)	83.33 (73.33 – 90.00)	83.33 (70.00 – 86.67)	81.66 (73.33 – 90.00)		
Min – Max	47 – 100	47 – 100	53 – 100		
Furcation Grade 1 [%] §					
Mean ± SD	14.73 ± 10.63	14.13 ± 10.07	15.33 ± 11.23	0.575^tt2^	Mean dif. CI [-5.4, 3]
Median (Q1 – Q3)	13.33 (6.67 – 23.33)	13.33 (6.67 – 20.00)	13.33 (6.67 – 23.33)		
Min – Max	0 – 43	0 – 37	0 – 43		
Furcation Grade 2 [%] §					
Mean ± SD	2.00 ± 3.964	2.66 ± 4.86	1.33 ± 2.69	0.094^tt2^	Mean dif. CI [-0.23, 2.9]
Median (Q1 – Q3)	0 (0 – 3.33)	0 (0 – 3.33)	0 (0 – 3.33)		
Min – Max	0 – 27	0 – 27	0 – 13		
Furcation Grade 3 [%] §					
Mean ± SD	2.23 ± 5.32	2.734 ± 6.12	1.73 ± 4.38	0.350^tt2^	Mean dif. CI [-1.1, 3.1]
Median (Q1 – Q3)	0 (0 – 0)	0 (0 – 0)	0 (0 – 0)		
Min – Max	0 – 27	0 – 27	0 – 20		
Tooth mobility Grade 0 [%] ¶					
Mean ± SD	87.72 ± 11.97	90.44 ± 9.29	85.00 ± 13.72	**0.023**^**tt2**^	Mean dif. CI [0.78, 10]
Median (Q1 – Q3)	89.06 (84.38 – 96.88)	92.18 (84.38 – 100)	87.50 (81.25 – 93.75)		
Min – Max	28 – 100	59 – 100	28 – 100		
Tooth mobility Grade 1 [%] ¶					
Mean ± SD	11.38 ± 11.14	8.56 ± 7.70	14.19 ± 13.25	**0.011**^**tt2**^	Mean dif. CI [-9.9, -1.3]
Median (Q1 – Q3)	9.380 (3.12 – 15.62)	6.25 (0 – 15.62)	12.50 (6.25 – 18.75)		
Min – Max	0 – 72	0 – 31	0 – 72		
Tooth mobility Grade 2 [%] ¶					
Mean ± SD	0.87 ± 2.48	1.0 ± 2.64	0.75 ± 2.33	0.616^tt2^	Mean dif. CI [-0.74, 1.2]
Median (Q1 – Q3)	0 (0 – 0)	0 (0 – 0)	0 (0 – 0)		
Min – Max	0 – 12	0 – 12	0 – 12		
Tooth mobility Grade 3 [%] ¶					
Mean ± SD	0.03 ± 0.31	0 ± 0	0.06 ± 0.44	0.322^tt2^	Mean dif. CI [-0.19, 0.06]
Median (Q1 – Q3)	0 (0 – 0)	0 (0 – 0)	0 (0 – 0)		
Min – Max	0 – 3.1	0 (0 – 0)	0 – 3.1		
Number of teeth in need of subgingival instrumentation *††*					
Mean ± SD	2.06 ± 2.14	1.92 ± 2.10	2.20 ± 2.19	0.515^tt2^	Mean dif. CI [-1.1, 0.57]
Median (Q1 – Q3)	1.0 (0 – 3.00)	1.0 (0 – 3.00)	2.0 (0 – 3.00)		
Min – Max	0 – 8	0 – 7	0 – 8		

Abbreviations: *n* number of participants, *SD* standard deviation, *Q* quartile, *Min* Minimum, *Max* Maximum, *CI* Confidence Interval, ^*tt2*^ Welch’s two-sample t-test, ^*chi2*^ Pearson's chi-squared test, † Ainamo, J., & Bay, I. (1975), ‡ O’Leary, T. J., Drake, R. B., & Naylor, J. E. (1972), § Hamp, S. E., Nyman, S., & Lindhe, J. (1975), ¶ Lindhe, J., & Nyman, S. (1977), †† teeth with probing pocket depths ≥ 5 mm or 4 mm + bleeding on probing. Significant values are in bold

### OHRQoL and experience with MRONJ

Overall, the OHIP-G14 sum score was 4.86 ± 6.14. Participants with history of ART assessed a statistically significantly lower OHRQoL with a sum score of 6.10 ± 6.76 compared to unexposed with 3.62 ± 5.22 (*p* = 0.043^tt2^). Correspondingly, differentiating between the seven sub-domains, ART-exposed participants evaluated overall lower OHRQoL compared to unexposed, albeit only statistically significantly (*p* = 0.016^tt2^) in the sub-domain “handicap”, in which participants were asked if, due to problems with their teeth, mouth, or dentures, their lives would have been “generally less satisfying” and they would have been “totally unable to function” (0.84 ± 1.39 versus 0.30 ± 0.68; Supplementary Figure 1).

Among the ten participants (10%) who reported the lowest OHRQoL with OHIP-G14 sum scores ranging from 15 to 24 (19.30 ± 3.53), eight had received antiresorptives and two had a history of MRONJ.

Out of the fifty participants under ART, 39 (78%) stated to be aware of their MRONJ-risk. 20% were sent to an oral check-up prior to the administration of antiresorptives and five participants (10%) stated they got teeth extracted as a precautionary measure before ART. Twenty participants (40%) reported having received information about oral preventive measures during ART, and 46 (92%) would have liked to receive additional oral preventive measures if only they had been available (Table 3).

**Table 3:** OHRQoL and experiences with ART

Variables	Total (*n* = 100)	Unexposed Group (*n* = 50)	ART-exposed Group (*n* = 50)	*p*-value	Confidence Interval
OHIP-G14 sum score					
Mean ± SD	4.86 ± 6.14	3.62 ± 5.22	6.10 ± 6.76	**0.043**^**tt2**^	Mean dif. CI [-4.9, -0.08]
Median (Q1 – Q3)	2.50 (0 – 7.00)	1.50 (0 – 5.00)	3.50 (1.00 – 9.00)		
Min – Max	0 – 24	0 – 24	0 – 24		
Awareness of MRONJ-risk					
No			11 (22%)		
Yes			39 (78%)		
Oral check-up prior to ART					
No			40 (80%)		
Yes			10 (20%)		
Precautionary tooth extraction before ART					
No			45 (90%)		
Yes			5 (10%)		
Information of oral preventive measures while receiving ART					
No			30 (60%)		
Yes			20 (40%)		
Willingness for further preventive measures while receiving ART					
No			4 (8%)		
Yes			46 (92%)		

Abbreviations: *n* number of participants, *SD* standard deviation, *Q* quartile, *Min* Minimum, *Max* Maximum, *CI* Confidence Interval, ^*tt2*^ Welch’s two-sample t-test, *MRONJ* medication-related osteonecrosis of the jaw. Significant values are in bold

### Regression analysis

The best linear model for the mean CAL at patient level revealed “pack years”, as the only statistically significant variable after stepwise variable selection using AIC (*p* = 0.0173). For two patients with one pack year difference, the mean CAL for the patient who had smoked more was 0.36% higher than for the patient who smoked less. The statistically significant variables in the final model for PPD are “PCR” (*p* = 0.0022) and “diabetes mellitus” (*p* = 0.0333). Consequently, for two patients with one percent difference in PCR, the mean PPD for the patient with the higher PCR was 0.19% higher than for the patient with the lower PCR. For a patient with diabetes mellitus the mean PPD was 6.73% higher than for a patient without diabetes. In the best model for BOP, again, the only statistically significant variable is “PCR” (*p* < 0.001), meaning for two patients with one percent difference in PCR, the square-root mean BOP for the patient with the higher PCR was by 0.0034 higher than for the patient with the lower PCR. In the final linear model for the OHIP-G14 sum score, the only statistically significant variable is “DMFS”. For two patients with a DMFS index difference of one, the square-root OHIP-G14 sum score for the patient with the higher DMFS was by 0.0194 higher than for the patient with the lower DMFS index (Table 4).

**Table 4:** Regression analysis

(A) Best linear model for the logarithmic mean clinical attachment level at patient level after stepwise variable selection using AIC (*n* = 100)				
Variable	Estimate	Lower 95% CI	Upper 95% CI	*p*-Value
ART-exposed Group	-0.0513	-0.1311	0.0284	0.2044
Age	0.0039	-7e-04	0.0086	0.0956
Diabetes mellitus: yes	-0.0018	-0.129	0.1253	0.9775
Smoking: pack years	0.0036	7e-04	0.0066	**0.0173**
(B) Best linear model for the logarithmic mean probing pocket depth at patient level after stepwise variable selection using AIC (*n* = 100)				
Variable	Estimate	Lower 95% CI	Upper 95% CI	*p*-Value
ART-exposed Group	-0.0343	-0.071	0.0025	0.0674
Age	-0.002	-0.0041	2e-04	0.0729
Diabetes mellitus: yes	0.0651	0.0053	0.125	**0.0333**
Smoking: pack years	3e-04	-0.0011	0.0016	0.6901
Plaque Control Record †	0.0019	7e-04	0.0031	**0.0022**
(C) Best linear model for the square root mean bleeding on probing at patient level after stepwise variable selection using AIC (*n* = 100)				
Variable	Estimate	Lower 95% CI	Upper 95% CI	*p*-Value
ART-exposed Group	-0.0155	-0.0727	0.0416	0.5909
Age	8e-04	-0.0025	0.0041	0.6291
Diabetes mellitus: yes	-0.0031	-0.0963	0.09	0.9467
Smoking: pack years	-7e-04	-0.0028	0.0015	0.541
Plaque Control Record **†**	0.0034	0.0015	0.0053	** < 0.001**
(D) Best linear model for the square root OHIP-G14 sum score at patient level after stepwise variable selection using AIC (*n* = 100)				
Variable	Estimate	Lower 95% CI	Upper 95% CI	*p*-Value
ART-exposed Group	0.4816	-0.0752	1.0384	0.0892
Age	-0.0317	-0.0644	9e-04	0.0567
Diabetes mellitus: yes	0.1821	-0.715	1.0792	0.6878
Smoking: pack years	-0.0103	-0.0312	0.0105	0.3278
DMFS-Index	0.0194	0.0058	0.0331	**0.0057**

Abbreviations: *CI* confidence *interval*, *†* O’Leary, T. J., Drake, R. B., & Naylor, J. E. (1972). Significant values are in bold

## Discussion

This cross-sectional study with group comparison exhibited similar dental and periodontal findings in patients of advanced age with and without ART involved in regular SPC. CAL along with PPD, BOP and furcation involvement showed no statistically significant differences between the two groups, whereas slight tooth mobility of grade 1 was more prevalent in the exposed compared to the unexposed group. These findings, e.g. mean PPD of 2.35 ± 0.24 mm, 8.61 ± 5.73 years after active periodontal therapy, are comparable to those of Matuliene et al. who showed eleven years after active periodontal therapy mean PPDs of 2.1 ± 0.4 mm [27]. Regression analysis showed that diabetes mellitus and a higher PCR was associated with a higher PPD, and a history of smoking (pack years) with a higher CAL as it is described in the literature [28].

DMFT and DMFS findings revealed no group differences in the total index, as well as regarding carious, missing or restored teeth and surfaces, respectively. The observed DMFT index of 18.17 ± 3.95 is comparable to the findings of the Fifth German Oral Health Study (DMS V) that showed a DMFT of 17.7 in seniors aged 65 to 74. The same applies for the DMFS of 67.43 ± 20.47 and a slightly higher index of 72.6 in DMS V [29].

OHRQoL was evaluated statistically significantly lower in patients under ART compared to unexposed. The investigation of OHRQoL by means of the OHIP-G14 as a widely used assessment tool, does not reflect the clinical oral status, but the individual’s perception of oral health and its impact on life. This multidimensional concept involves biopsychosocial aspects related to oral health and can be influenced by cultural context and age [30]. In these cases, influential factors could be the knowledge of the MRONJ-risk and a stronger emphasis on oral health in particular on periodontal stability. The OHIP-G14 sum score of 3.62 ± 5.22 in the unexposed group however, is similar to other investigations of OHRQoL in SPC. Greatz et al. showed an OHIP-G14 sum score of 3.7 ± 5.6 and Sonnenschein et al. an OHIP-G14 sum score of 3.64 ± 5.41 [31, 32].

Evidence on periodontal therapy during ART is sparse and to our knowledge, there is no scientific data on patients under ART in periodontal maintenance. However, the topic is of high clinical and scientific importance. Periodontitis is one of the most common chronic inflammatory diseases worldwide and underlying systemic diseases for ART such as osteoporosis or cancer are highly prevalent [33]. In the context of an aging society, a further increase in prevalence of all three can be assumed and concurrently an increase of MRONJ as a side effect of ART must be expected, particularly as a well-documented consequence of infectious conditions in the alveolar bone such as periodontitis [34]. In the present study, three out of fifty periodontally compromised patients under ART developed MRONJ due to periodontal reasons and one due to endodontic/restorative reasons. Wick et al. identified periodontal disease with an odds ratio of 2.46 (1.12–5.40, *p* = 0.026) to increase MRONJ onset in patients treated with DNO [6]. Lorenzo‐Pouso et al. confirmed a risk ratio of 2.75 (1.67–4.52) of periodontal disease in MRONJ‐affected sites compared with at‐risk but non‐affected patients [35]. Therefore, this subject is of high importance now and moreover in the future for periodontally compromised patients as well as for dentists and maxillofacial surgeons in the decision-making process, treatment planning, but also therapy itself. Precautions, such as administering antibiotics during invasive dental procedures should be taken. Further investigations on specific treatment protocols, also specifically for active and SPC are necessary [12, 14, 15, 36].

The varying MRONJ-risk relating to the underlying systemic disease being the reason for ART, but also the duration and dosage of medication should be considered. Lower BPs concentrations and longer treatment intervals are usually established in patients with osteoporosis compared with patients in whom cancer-related antiresorptives are used and higher concentrations and more frequent doses are required [1–3]. The prevalence of MRONJ is reported to be 0–0.5% in osteoporotic patients with a low risk profile [7, 37]. In patients on cancer-related ART, it is estimated to be 1–21% with a high-risk profile [7, 38–40]. In this observation, the overall prevalence of MRONJ is 8% and there is an indication of different risk profiles, as three out of fifty participants under ART, who developed MRONJ due do periodontal reasons, had cancer as underlying disease. As evidenced by the patient records, the two patients who developed MRONJ during SPC were informed of their MRONJ-risk in the past, and tooth extractions of periodontally questionable teeth were recommended, but the patients refused. It can be hypothesized that especially these high-risk patients could benefit from early extractions of periodontally severely affected teeth, additional precautions such as the administration of antibiotics and intensified preventive measures, while patients with osteoporosis as an underlying disease might be at lower risk. However, further prospective studies on the different MRONJ-risk profiles with larger sample sizes of patients with cancer are needed to draw reliable conclusions on that matter. The findings of this study provide indications on different MRONJ-risk profiles during SPC but definitive conclusions cannot be derived because 1) history of MRONJ was investigated descriptively, 2) patients with different risk profiles were not equally represented and 3) further analysis of MRONJ-affected patients revealed non-adherence. Also, a specialized university environment with close interaction with maxillofacial surgeons is not ubiquitous. This limits the generalizability of the findings. On the other side, this patient sample is representative of an elderly patient population during SPC in Germany. Since the clinical examination was part of a routine SPC session, blinding of the examiner to patient group allocation was not possible. Further investigations on the influence of ART on periodontal treatment over time as well as vice versa periodontal treatment on the risk or prevention of the development of MRONJ considering different risk profiles are necessary.

### Conclusion

Within the limitations of this study, elderly patients under antiresorptive medication in regular SPC demonstrated similar clinical periodontal and dental status, but lower OHRQoL compared to unexposed. Further studies on different MRONJ-risk profiles with larger sample sizes of patients with cancer are needed to draw reliable conclusions on the MRONJ-risk during periodontal therapy. But it can be hypothesized that especially these high-risk patients could benefit from intensified preventive care while ostoporotic patients could be at lower risk. Patient awareness of the MRONJ-risk and appropriate preventive measures should be ensured.

### Supplementary Information

Below is the link to the electronic supplementary material.

Supplementary file1. Supplementary Figure 1. Bar plot and descriptive statistics of the OHIP-G-14 sub-domains. Abbreviations: SD = standard deviation, Q = quartile, Min = Minimum, Max = Maximum (TIFF 16886 KB)

## Data Availability

All data generated or analysed during this study are included in this article.
