# Supplementary figures and images for: Oral health conditions in patients under antiresorptive therapy are comparable to unexposed during supportive periodontal care
Source: Clin Oral Investig. 2023 Sep 15;27(11):6523–36. doi: 10.1007/s00784-023-05257-y (PMC10630227; doi:10.1007/s00784-023-05257-y)

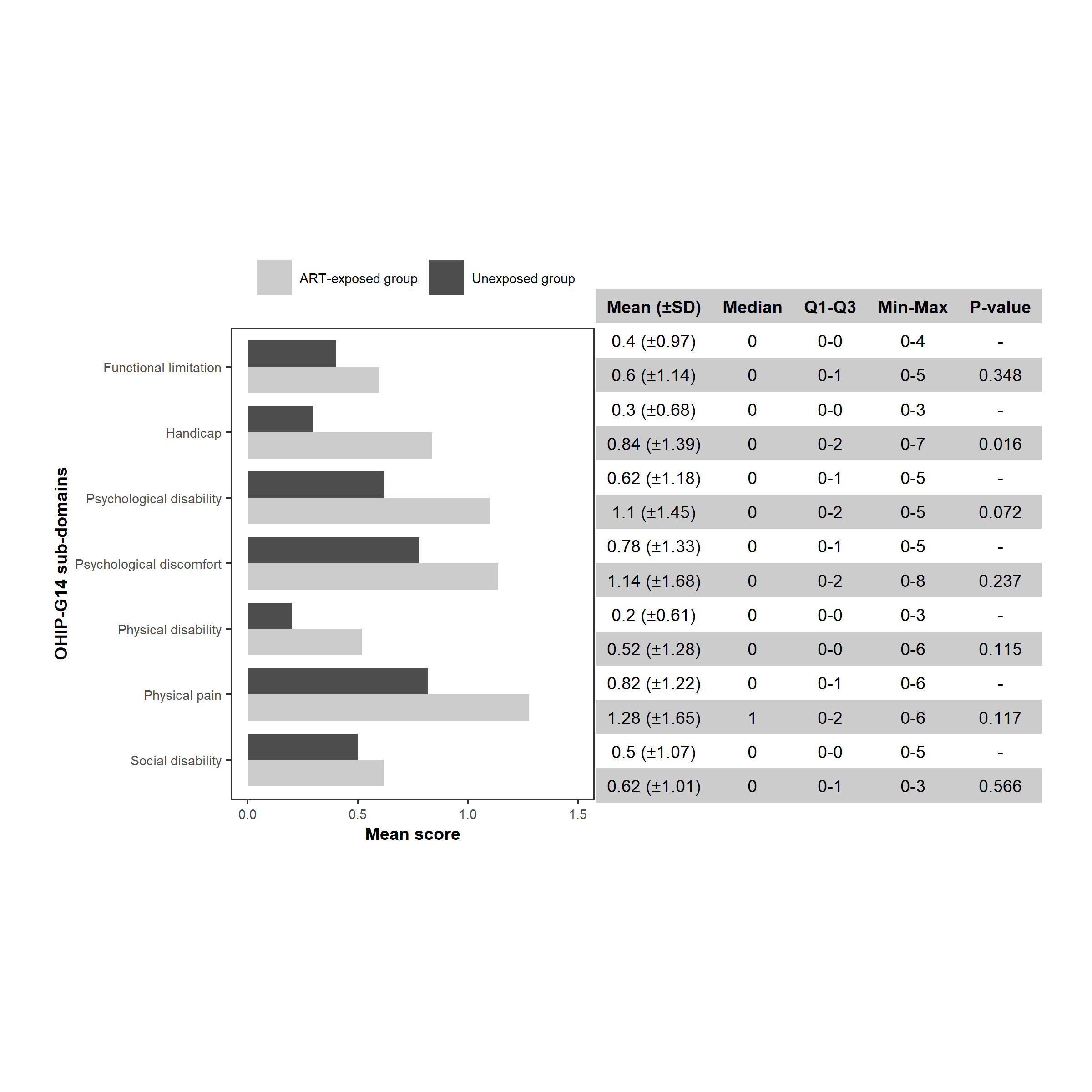

Supplement: Supplementary file 1 — Supplementary file1. Supplementary Figure 1. Bar plot and descriptive statistics of the OHIP-G-14 sub-domains. Abbreviations: SD = standard deviation, Q = quartile, Min = Minimum, Max = Maximum (TIFF 16886 KB) [file 784_2023_5257_MOESM1_ESM.tiff]
